# Decomposition of years of life lost due to premature death (YLL): a method for spatial and temporal comparative assessment

**DOI:** 10.1186/s13690-020-00472-5

**Published:** 2020-10-06

**Authors:** Nitya Saxena, Deepak Sethia

**Affiliations:** Department of Economics, Indian Institute of Management, Indore, India

**Keywords:** Disease burden, DALY, YLL, Decomposition, Methodology, Health

## Abstract

**Background:**

Acceptance of Disability-Adjusted Life Year (DALY) as a measure of health summary and progression has increased over the years, which in turn has instigated comparative analysis studies of DALY across time and geography. Thus, it is important to explore methodological underpinnings of comparative analysis.

**Results:**

A crude comparison of disease burden across time or space may mislead the interpretation of the health system’s performance because the quantum and pattern of DALY can be influenced by the age structure of the society. A significant proportion of this burden is due to the Years of Life Lost (YLL) component. The paper proposes a mathematical exposition to decompose the change in YLL over time or region into burden attributed to a) population age structure, b) death rate, and c) age at death gradient.

**Conclusion:**

We reasoned that the death rate and age at death burden gradient signify the real contribution of the health systems. Hence, the method of decomposition can be utilized to measure the health service progression of a region in real terms.

## Background

Globally, each country is striving to achieve better health for its citizens. In order to measure ‘betterment’ different methods have been adopted to attain allocative efficiency of limited resources. To reach allocative efficiency, policies driven solely by mortality rate are not enough as they are not comprehensive to account for morbidity, disease category, cost-effectiveness, health perception, and decision making [1]. The need for the comprehensive measure was felt to account for these limitations, and hence population health summary measures like Disability Adjusted Life Years (DALY) took shape. The summary measures are techniques to represent morbidity and mortality in a single number [2].

DALY represents a loss of time due to disability caused by a particular disease [3]. Since it is mapped to a particular disease condition, it deemed suitable for quantifying the burden of disease and injury, cost-effectiveness, and resource allocation [4, 5]. DALY is composed of two components: Years of life lost due to premature death (YLL) and Years of life lived with disability (YLD). YLL reflects life lost between the age of death and life expectancy at the age of death, while YLD represents life loss due to morbidity.

Subsequently, DALY methodology is adopted by WHO for Global Burden of Disease study and has been advocated by the international body for global health status benchmarking.

One set of studies focused on DALY calculation for a particular year and region for a set of diseases and risk factors (see [6–13]). In recent years with the availability of chronological data, another set of studies engaged in the spatial and temporal comparison of DALY. With an increasing number of countries enabled to carry out DALY comparative analysis across time and space [14–16], it is apt time to engage in the comparative analysis methodology discourse.

With this background, this paper proposes a method for comparison by decomposition of the burden of premature death into three attributes: a) population age structure, b) death rate, and c) age at death. While doing so, we empirically argue the use of region-specific population proportion instead of the global level average population in disease burden calculation. The method can be extended to decompose YLD as well in the future. Section 2 discusses the rationale for this decomposition. Section 3 details the process of decomposition and provide mathematical exposition. As shown in section 4, the decomposition method can be utilized to measure the health service progression of a region across different time and space. Section 5 summarizes and discusses the intuitive relevance of decomposed parameters in case of communicable, non-communicable, and injuries.

### The rationale for the decomposition of disease burden into: age-structure, death rate and age at death

To measure the progress of health systems, DALY has become an internationally accepted parameter. It is often used to measure the difference in disease burden over time or across regions (or space). However, a crude comparison of DALY across time or space may mislead the interpretation on the performance of the health system because the quantum and pattern of DALY can be influenced by the age structure of the society. For example, a developed country may have a high NCD (non-communicable disease) burden due to population aging despite the reduction in age-specific death rate. Similarly, a developing nation may have a lower NCD burden majorly due to young age population.

**Fig. 1:**
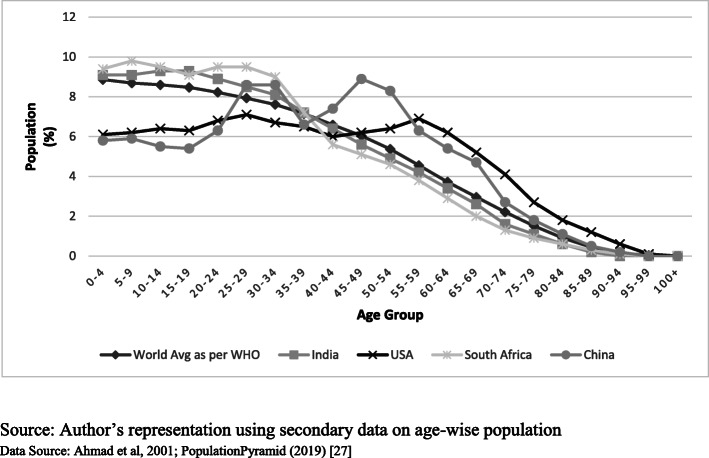
Age Structure of World average (2000–2025) vs other regions (2018). Source: Author’s representation using secondary data on age-wise population. Data Source: Ahmad et al., 2001 [18]; PopulationPyramid (2019) [17]

To address this issue, the WHO has proposed age standardization of the world population [18]. But as depicted in Fig. 1, this standardization does not reflect the age structure of several economies undergoing a demographic transition. The issue becomes more significant for cross-country comparison where the global population structure may not have much relevance for the nations under consideration. WHO report also acknowledges this issue of non-representativeness [18].

Further, the age-structure becomes more diverse if the sub-national population is considered. For example, a society with a high share of infants in the population is likely to have different diseases inflecting its members compared to a society that has a high share of the old age population. Given that deaths and diseases largely strike at younger and older age populations, a society with a high share of the middle-age population is likely to have a lower level of disease burden for a given level of the health system.

Hence, to stick to the motive of calculating DALY that is truly experienced by regions, we propose to use region-specific age structure and later attribute its effect during comparative analysis. This approach is likely to improve the robustness of DALY as a method for global health status benchmarking.

Previous studies have also approached comparison in a similar fashion with certain differences. In an inter-temporal comparison study, Murray et al. (2012) [14] have controlled for population structure while comparing the Global Burden of Disease results for 1990 and 2010. The basic implicit approach is to keep population structure constant over two times, and then evaluate the change in the death rate to analyze the impact of health improvements. However, a similar method study could not be found for spatial comparison where the regional population structure of a country is allowed to influence their own disease burden. Ghosh and Arokiasamy (2009) [19] control for population structure while comparing disease burden for Indian states by applying all India age structure for all states on state-specific mortality rates but this paper confuses disease burden with mortality, while the former is supposed to convey years of life lost rather than the number of lives lost. Further, both the papers have implicitly controlled for age structure for their purpose, without providing a generalized mathematical decomposition of the change in burden due to age structure and death rate across time and region. This paper provides this exposition by means of mathematical decomposition equations.

The decomposition formula helps to remove the possible biases due to the choice of *age structure,* which confounds the contribution of change in death rate towards the reduction of burden. It is the contribution of *change in death rate* in influencing disease burden, which can rightly be considered as the contribution of health systems. Additionally, within contribution due to *change in death rate*, the paper further identifies the possibility to separate out the contribution of change in aggregate death rate vs change in death rate across age groups. As discussed in detail in the next section, this step will further segregate the effect of death rate and age at death on disease burden. From now on ‘change in burden due to death rate’, ‘change in burden due to age structure’ and ‘change in burden due to age at death’ will be referred as ‘death rate gradient,’ ‘age structure gradient,’ and ‘age at death gradient’ respectively.

### Decomposition of YLL for inter-temporal and spatial comparison

For decomposition, the YLL component of disease burden is considered as we are attempting to observe the progress made in delaying the age of death. With certain modifications, the same method can be extended to YLD as well, which can be considered as an exercise for the future.

Disease burden calculated with the DALY approach essentially captures the years of life lost (YLL), which, by definition, would be greater for death that takes place at an earlier age. YLL caused by death in a particular year can be measured as:

1 $$ YL{L}_{abst}={\sum}_i^N{D}_{it}L{E}_i $$

Where i = age/age group.

D: number of deaths taking place at the given age.

LE: conditional life expectancy at the given age.

Subscript ‘abs’ refers to the absolute gross number of YLL.

‘t’ refers to a year (or region) for which number is being calculated.

The above measure provides an absolute quantum of YLL for a society. However, we need to control for the size of the population to engage in inter-temporal or spatial comparison. This can simply be done by dividing the YLL by population (*P*_*t*_) at that instance (time or region). A further multiplication with 1000 can be done to make number per 1000, as Infant Mortality Rate^1^ and other health indicators are represented at this level. Subscript ‘abs’ is removed from YLL to make it as a rate per 1000 in the time (or region) ‘t’.

2 $$ {YLL}_t={\sum}_i\frac{D_{it}{LE}_i}{P_t}\ast 1000 $$

Eq. (3) is obtained by multiplying and dividing by P_it_ (age-group ‘i’ population for instance ‘t’)

3 $$ {YLL}_t={\sum}_i\frac{P_{it}{D}_{it}{LE}_i}{P_{it}{P}_t}\ast 1000 $$

Let $DRi=\frac{D_i}{P_i}$ *1000 which reflects death rate per 1000 for given age-group `i`,

and $PWi=\frac{P_i}{P}$ which reflects the share/weight of the particular age group in the population.

Then,

4 $$ {YLL}_t={\sum}_{i=1}^NP{W}_{it}\ast {LE}_i\ast D{R}_{it} $$

It can be noted that subscript ‘t’ has been used at all other places except for LE. This is because, for any comparison of YLL over space or time, years lost due to death at a particular age are considered exactly the same out of ethical considerations of valuing life at the same scale across space and time. This is in line with the methodology proposed by Murray (1994) [4] and reasoned as a measure of disease burden combined with under-development by Anand and Hanson (1997) [20].

Next, mathematically, the overall change in YLL between time 0 and 1 (or region 0 and 1) can be calculated as follows:

5 $$ \Delta \ YLL={\sum}_{i=1}^NP{W}_{i1}\ast {LE}_i\ast D{R}_{i1}-{\sum}_{i=1}^NP{W}_{io}\ast {LE}_i\ast D{R}_{i0} $$

It can be noted from equation (5) that change in YLL can take place due to either change in a) age structure (*PW*_*io*_ *to PW*_*i*1_) or b) death rate (*DR*_*i*0_ *to DR*_*i*1_). Age structure implies the proportion of individuals in each age group, and the death rate represents the respective proportion of deaths.

It is the contribution of death rate gradient in influencing YLL, which can rightly be considered as the contribution of health systems. For this, the impact of the age structure gradient should be separated from the overall change in YLL. Other than impacting overall YLL, age structure gradient can also influence the distribution of YLL across different diseases.

To control for the contribution of age structure in changing the YLL equation (5) is decomposed into two components, namely the population age structure burden gradient and the death rate burden gradient (equation 6).

6 $$ \Delta \ YL L=\Delta \ YL{L}_{age}+\Delta \ YL{L}_{death} $$

Based on the choice of weight, the decomposition can follow either partial or total contribution approach. While decomposing with the partial contribution, weights used are of base instance ‘0’ while in the total contribution, weights are of instance ‘1’. Here, a parallel comparison can be drawn from Laspeyres and Paasche Indices in economics where former use base period price or quantity and later uses current period price or quantity as weights [21]. Subsequently, an averaging, comparable to the fisher index in this context, is proposed to combine the results of two decomposition approaches.

$$ Partial\ Contribution\ approach\ \left( weights\ of\ instance^{\prime }0^{\prime}\right): $$

7 $$ \Delta \ YL{L}_{age}={\sum}_{i=1}^NP{W}_{i1}\ast {LE}_i\ast D{R}_{i0}-{\sum}_{i=1}^NP{W}_{io}\ast {LE}_i\ast D{R}_{i0} $$

and

8 $$ \Delta \ YL{L}_{death}={\sum}_{i=1}^NP{W}_{io}\ast {LE}_i\ast D{R}_{i1}-{\sum}_{i=1}^NP{W}_{io}\ast {LE}_i\ast D{R}_{i0} $$

In equation (7), *∆ YLL*_*age*_ shows that for the instance ‘0’ (time or space), the population structure was *PW*_*io*_ - which has changed to *PW*_*i*1_. Hence, keeping the death rate the same, we can separate the contribution of population structure over time (or region). Equation (8) shows *∆ YLL*_*death*_, which is calculated while keeping the population structure the same, but allowing the death rate to change. However, another scenario is also possible using weights of instance ‘1’ that yields equation (9, 10):

$$ Total\ Contribution\ approach\left( weights\ of\ instance^{\prime }{1}^{\prime}\right): $$

9 $$ \Delta \ YL{L}_{age}={\sum}_{i=1}^NP{W}_{i1}\ast {LE}_i\ast D{R}_{i1}-{\sum}_{i=1}^NP{W}_{io}\ast {LE}_i\ast D{R}_{i1} $$

and

10 $$ \Delta \ YL{L}_{death}={\sum}_{i=1}^NP{W}_{i1}\ast {LE}_i\ast D{R}_{i1}-{\sum}_{i=1}^NP{W}_{i1}\ast {LE}_i\ast D{R}_{i0} $$

Notice the change in weights of *DR* and *PW* for equation (9) and (10), respectively, as compared to the previous set. To reiterate, equation (7, 9) both represent the change in burden due to age structure, but equation (7) utilizes the weight of instance ‘0’(DR_0_) while equation (9) utilizes the weight of instance ‘1’ (DR_1_). Similarly, in death rate differential equation (8) weights are of instance ‘0’ (PW_0_), and in equation (10) weights are of instance ‘1’ (PW_1_). Note that equation 6 is decomposed into equations 7 and 8 (partial contribution) or equations 9 and 10 (total contribution). However, when they are added back to obtain equation (6), we end up with a ‘residual’ term, which is discussed below.

Both sets of the equation are meaningful but suffer from bias in estimation which needs to be averaged out. Let’s presume that society is experiencing demographic transition, with the rise in the share of the old-age population and a corresponding decline in the share of the younger age population. This assumption fairly characterizes most developing countries. If, for example, the first set (equation 7 and 8) is used for temporal comparison, then equation (8) while measuring the contribution of change in death rate, uses population structure of year ‘0’ which had a high share of younger age population. By providing higher weight to the younger age population, equation (8) will overestimate the reduction in YLL due to a fall in death rate. Similarly, the impact of age structure on YLL reduction is also overestimated as the year ‘0’ is likely to have a relatively higher incidence of childhood mortality, which has seen a decline. It can be seen that the case of overestimation will be reversed for the second set of the equation. By using lower weight for younger age group, equation (10) underestimates the contribution of the declining rate of childhood mortality in overall YLL reduction. In this context, the partial contribution approach overestimates, and the total contribution approach underestimates the contribution from different components.

The magnitude by which the two approaches over- or underestimate the results is quoted as *‘residual’*(in literature), which prevents decomposed values to add up to the original. The residual arising from both the methods are equal in magnitude and opposite in direction [22]. To resolve the issue of residual, the paper follows a solution akin to the fisher price index, i.e., averaging the decomposed values calculated using both the methods [22, 23]. An empirical example elaborates it in section 4.

The impact of the death rate in *∆ YLL*_*death*_ based on equation (8) is considered as a key result in Murray et al. (2012). However, the impact of the death rate can further be decomposed into a change in overall death rate and change in age at which death took place. Health interventions can reduce the death rate leading to a reduction in YLL. However, the fall in death rate may not be even across age groups. Late age deaths can result in falling death rates in young age-group and rising death rates in old age-groups. Even this case will result in YLL reduction despite the overall death rate being the same.

**Fig. 2:**
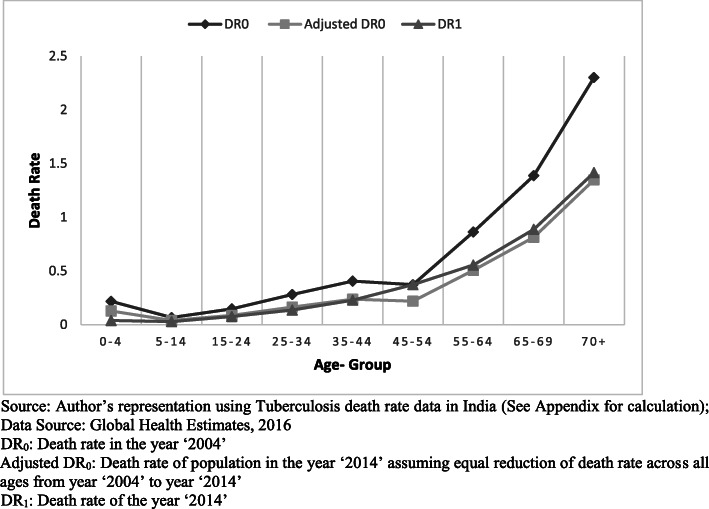
Stepwise change in Death Rate and Age at Death graph

Uneven reduction in death rate across age groups needs to be considered separately from the overall change in population death rate. It can be understood as an impact of “keeping the overall age structure and overall death rate constant but allowing age at death to change.” The process will yield fall (or rise) in burden due to rise (or fall) in age at death. Let’s understand the utility of this method with a real-time scenario of the temporal decomposition of the tuberculosis burden. India ranks first in the world ranking for tuberculosis burden [24]. In Fig. 2 (see Additional file 1), DR_0_ represents death rate in the year ‘2004’, DR_1_ is death rate in the year ‘2014’ for different age groups while ‘Adjusted DR_0_’ represents age-specific death rate in a hypothetical society if same death rate reduction is achieved across all age groups. But it can be observed from the graph that there is inequality in death rate reduction achieved for different age groups. Substantial reduction in death rate is achieved between age 5 and 70 except for age group 45–54. The proposed method allows capturing this inequality in a single number, i.e., *age at death burden gradient*.

Mathematically, equation (8) or (10) can be further decomposed into burden change due to age at death and death rate (equation 11). Again, it can be done in two ways, partial contribution (equation 12 and 14) and total contribution approach (equation 13 and 15) and averaged out later.

11 $$ \Delta \ YL{L}_{death}=\Delta \ YL{L}_{age\  at\  death}+\Delta \ YL{L}_{death\ rate} $$

$$ Partial\ Contribution\ approach\left( weights\ of\ instance^{\prime }0^{\prime}\right): $$

12 $$ \Delta \ YL{L}_{age\  at\  death}={\sum}_{i=1}^NP{W}_{io}\ast {LE}_i\ast D{R}_{i1}-{\sum}_{i=1}^NP{W}_{io}\ast {LE}_i\ast D{R}_{i0}\ast \frac{\sum_{i=1}^NP{W}_{i0}\ast {DR}_{i1}}{\sum_{i=1}^NP{W}_{i0}\ast {DR}_{i0}} $$

$$ Total\ Contribution\ Approach\left( weights\ of\ instance^{\prime }1^{\prime}\right): $$

13 $$ \Delta \ YL{L}_{age\  at\  death}={\sum}_{i=1}^NP{W}_{i1}\ast {LE}_i\ast D{R}_{i1}-{\sum}_{i=1}^NP{W}_{i1}\ast {LE}_i\ast D{\mathrm{R}}_{i0}\ast \frac{\sum_{i=1}^NP{W}_{i1}\ast {DR}_{i1}}{\sum_{i=1}^NP{W}_{i1}\ast {DR}_{i0}} $$

Here 1st term on the right-hand side of equation (12) and (13) represents aggregate *burden gradient* of time (or region) ‘1’ weighted on the age structure of time (or region) ‘0’ and ‘1’ respectively. The second term of the right-hand side reflects the aggregate burden gradient that would have occurred if the distribution of deaths across age groups could be hypothetically matched to the time (or region) ‘0’ while keeping overall death rate of time (or region) ‘1’. Difference between the two terms can be referred to as the impact of age at death gradient on aggregate burden gradient. Note that in the 2nd component of the right-hand side of equation 12 (and respectively 13), the overall death rate (${\sum}_{i=1}^NP{W}_{io}\ast D{R}_{i0}\ast \frac{\sum_{i=1}^NP{W}_{i0}\ast {DR}_{i1}}{\sum_{i=1}^NP{W}_{i0}\ast {DR}_{i0}}$) will remain the same as in the 1st component of the right-hand side (${\sum}_{i=1}^NP{W}_{i0}\ast {DR}_{i1}$). Hence, the difference in the YLL for two sides arises because of differences in the age at which death takes place.

An (extreme) example would better elaborate on the meaning and significance of this factor. Presume that we have exactly the same age structure over two-time periods, giving us exactly the same PW for both times ‘0’ and ‘1’. Overall the death rate for both periods 0 and 1 is also exactly the same at DR_x_. However, in period ‘0’, most of the deaths are concentrated at a younger age group, but in period ‘1,’ these deaths are delayed and takes place in higher age groups. Hence, this will lead to a fall in YLL, which can be attributed to the rise in age at death rather than the fall in death rate. In practice, one can expect that age at death for NCDs should rise with better health systems, which would result in a fall in YLL. On the other hand, in the case of neonatal mortality, age at death would, by definition, remains the same over two-time periods. Hence, the entire fall due to death pattern can only come from a change in death rates. This can be captured as:

$$ Partial\ Contribution\ Approach\left( weights\ of\ instance^{\prime }0^{\prime}\right):: $$

14 $$ \Delta \ YL{L}_{death\ rate}={\sum}_{i=1}^NP{W}_{io}\ast {LE}_i\ast D{R}_{i0}\ast \frac{\sum_{i=1}^NP{W}_{i0}\ast {D}_{i1}}{\sum_{i=1}^NP{W}_{i0}\ast {D}_{i0}}-{\sum}_{i=1}^NP{W}_{io}\ast {LE}_i\ast D{R}_{i0} $$

T *otal Contribution Approach*(*weights of instance* ′ 1′) : :

15 $$ \Delta \ YL{L}_{death\ rate}={\sum}_{i=1}^NP{W}_{i1}\ast {LE}_i\ast D{R}_{i0}\ast \frac{\sum_{i=1}^NP{W}_{i1}\ast {D}_{i1}}{\sum_{i=1}^NP{W}_{i1}\ast {D}_{i0}}-{\sum}_{i=1}^NP{W}_{i1}\ast {LE}_i\ast D{R}_{i0} $$

As mentioned previously, the overall death rate in the 1st component of the right-hand side in equation (14) is equivalent to ${\sum}_{i=1}^NP{W}_{i0}\ast {D}_{i1}$, which differs from the 2nd component in the instance of the death rate. However, the percentage distribution of deaths across various age groups remains the same in both cases. Hence, change in YLL can entirely be attributed to the fall in death rate. It may be noted that a negative value of *death rate gradient* as well as *age at death gradient* is desirable as it signifies a lower death rate and delayed age at death.

### The empirical illustration of temporal and spatial decomposition

Mozambique, a Sub-Saharan African country, once had a substantial communicable, maternal, neonatal, and nutritional mortality rate in the region [25], which has been reduced significantly in recent decades. Table 1 illustrates decomposed temporal estimates of change in YLL from 2000 to 2016 for females in Mozambique (see Additional file 2). The data has been taken from YLL global health estimates by IHME GHDx [26, 27]. The decomposition via total and partial approach yields residuals, which has been averaged out to obtain residue-free estimates. The decomposed values highlight the major cause of YLL reduction as death rate gradient (76.71%) followed by age structure gradient (16.23%) and age at death gradient (7.06%). High death rate gradient indicates the key impact of the improved healthcare system in the region. Note that the negative sign implies a reduction in burden.

**Table 1:** Temporal comparison of the change in YLL burden between 2000 and 2016 for communicable, maternal, neonatal, and nutritional mortality in Mozambique 2016 (in years per thousand population)

δ YLL absolute (years per 1000 population)	− 367.69			
YLL 2000 (years per 1000 population)	691.90 at 95% CI [631.47–759.60]			
YLL 2016 (years per 1000 population)	324.21 at 95% CI [278.26–378.15]			
DECOMPOSED VALUES (years per 1000 population)				
	Residual	ΔAS^*^	ΔDR^*^	ΔDA^*^
TOTAL Contribution	− 50.92	− 34.21	− 258.45	−24.11
PARTIAL Contribution	50.92	− 85.13	− 305.65	−27.83
Residue free estimate	**0.00**	**−59.67**	**− 282.05**	**−25.97**
Contribution in δ YLL (%)		**16.23%**	**76.71%**	**7.06%**

YLL Burden gradient due to – *ΔAS* Age Structure, *ΔDR* Death Rate, *ΔDA* Age at Death.

(See Additional file 3 for calculation)

Similarly, Table 2 presents the spatial comparison of India and one of its states (undivided Andhra Pradesh) for tuberculosis in the year 2016 (see Additional file 3). The state has a lower absolute YLL burden by 4.56 years per thousand populations as compared to the national average.

**Table 2:** Spatial comparison of India and one of its state for tuberculosis YLL burden in 2016 (in years per thousand population

δ YLL absolute (years per 1000 population)	−4.56			
YLL India 2016 (years per 1000 population)	11.19 at 95% CI [10.48–11.90]			
YLL AP 2016 (years per 1000 population)	6.63 at 95% CI [5.24–8.62]			
DECOMPOSED VALUES (years per 1000 population)				
	Residual	ΔAS^a^	ΔDR^a^	ΔDA^a^
TOTAL Contribution	0.47	0.75	−5.08	−0.70
PARTIAL Contribution	−0.47	1.22	−4.63	−0.67
Residue free estimate	**0.00**	**0.98**	**−4.86**	**−0.69**
Contribution in δ YLL (%)		**−21.59%**	**106.50%**	**15.09%**

^a^YLL burden gradient due to- *ΔAS* Age Structure, *ΔDR* Death Rate, *ΔDA* Age at Death

However, the decomposition analysis enabled to realize better performance of state against country average is due to lower death rate and late age-at-death and not due to age structure. The state’s burden is 0.98 YLL per 1000 population higher (21.59% higher) than the national average due to age structure gradient but has lower burden due to death rate and age at death gradient (106.50 and 15.09% lower respectively). Negative death rate and age at death gradient imply better performance of State as compared to the national average.

(See Additional file 3 for calculation)

## Discussion and conclusion

Acceptance of DALY as a measure of health summary and progression has increased over the years, which in turn has instigated comparative analysis studies of DALY across time and geography. With this background, the paper introduced mathematical equations for temporal and spatial comparison and conceptualized the decomposition of the change in premature death burden into a) population age structure, b) death rate, and c) age-at-death.

The segregation of burden gradient due to population age structure removes the confounding effect of the region’s demography and enables fair assessment. With several economies facing demographic transition, a world population average may not reflect the actual burden. Hence a better method is to use the region’s population structure but separate out its effect during the comparative analysis. Next, the remaining value is further decomposed into death rate and age at death burden gradient, which can truly be considered as the contributions of the healthcare system.

Decomposition into death rate and age structure seems more relevant in the case of communicable diseases as the age-at-death gradient is not very significant. This is quite intuitive as death by communicable diseases usually occurs at a young age; hence its burden is likely to decline faster with age structure transition. In the case of non-communicable disease, age-structure and age-at-death burden gradient are likely to bear an opposite effect on the burden gradient. As the region’s population transits to aging, age structure burden gradient will assume a positive value. In this scenario, the death rate and age at death burden gradients will signify the `real contributions of the health systems. For injuries like road accidents and self-harm, death rate and age at death burden gradients can be used as a key parameter in policy targeting.

The decomposition method can be considered as an improvement over the existing methodology for two reasons. First, it allows and enables the use of local population structure in burden calculation, thus keeping the estimates close to the actual. Second, the decomposition method allows component-wise comparison between time-periods or regions, thus removing confounding effects and enabling better knowledge translation. Hence, the method of decomposition, which the study proposes, can be utilized to measure the health service progression of a region in terms of age at death, death rate, and age structure.

### Limitations and future work

The empircial illustrations provided in the paper are based on the existing disease burden estimates from the IHME GDBx database. It is desirable to estimate the confidence interval of Δ YLL and its components to understand the uncertainty in the proposed decomposition. While the IHME GDBx database provides estimates of the confidence intervals for estimates of YLL and number of deaths, we are unable to get details on the underlying statistical distribution involved in constructing the confidence interval. In the absence of the required details, we are unable to provide confidence intervals for the decomposed estimates given the empirical illustration. However, it should also be noted that the unavailability of the confidence interval in the empirical illustration does not have any impact on the validity of the proposed methodology for the decomposition of Δ YLL.

The proposed method uses a single life-expectancy benchmark for temporal (or spatial) comparison, which can be modified further to include provision for local life expectancy for accurate resource allocation decisions at the local level.

Further, the scope of the paper is limited to decomposition, which should be mapped to respective cost studies for resource allocation in the future. The decomposition method proposed is for YLL, a significant component of DALY, which can and should be extended to Years of Life lived with Disability (YLD) in the future for comprehensiveness.

## Supplementary information

**Additional file 1.** - Guideline to run the code. Figure 1: .py file

**Additional file 2.** Fig. 2: Excel sheet containing description, calculations and data to reproduce Fig. 2. Additional File 2 -Fig. 2: Python code for generation of Fig. 2. Additional File 2 -Deaths: Python code input files. Additional File 2 -POP: Python code input files. Additional File 2 Guideline to run the code

**Additional file 3.** Table 1 Excel file contains data and calculation of Table 1. It describes method for temporal decomposition of YLL between 2000 to 2016 in Mozambique for communicable, neonatal, maternal and nutritional deaths. Additional File 3 - Table 2: Excel file contains data and calculation of Table 2. It describes method for spatial decomposition of YLL between Indian and its State in 2016 for tuberculosis burden.. Additional File 3 - yll_decomposition: Python code to carry out decomposition. Additional File 3 - input data for Table 1 - POP: Input files to python code . Additional File 3 - input data for Table 1 – Deaths: Input files to python code. Additional File 3 - input data for Table 2 - POP: Input files to python code. Additional File 3 - input data for Table 2 – Deaths: Input files to python code. Additional File 3 - Guideline to run the code

## Data Availability

Additional files have been provided to allow reproduction and verification of results. The dataset(s) supporting the conclusions of this article is (are) included within the article (and its additional file(s)). Wherever applicable, public data source and additional file reference has been provided.

## Abbreviations

DALY: Disability Adjusted Life Years
GBD: Global Burden of Disease
GHDx: Global Health Data Exchange
IHME: Institute for Health Metrics and Evaluation
NCD: Non-Communicable Diseases
WHO: World Health Organization
YLD: Years Lost due to Disability
YLL: Years of life lost due to premature mortality

## Footnotes

1 The infant mortality rate is the number of deaths under one year of age occurring among the live births in a given geographical area during a given year, per 1000 live births occurring among the population of the given geographical area during the same year.

## References

[1] Gold MR, Stevenson D, Fryback DG (2002). HALYS and QALYS and DALYS, oh my: similarities and differences in summary measures of population health. Annu Rev Public Health.

[2] Molla MT, Wagener DK, Madans JH. Summary measures of population health: methods for calculating healthy life expectancy. Healthy People 2010 Stat Notes. 2001;(21):1–11. 10.1037/e583762012-001. 10.1037/e583762012-001 11676467

[3] Sassi F (2006). Calculating QALYs, comparing QALY and DALY calculations. Health Policy Plan.

[4] Murray CJ (1994). Quantifying the burden of disease: the technical basis for disability-adjusted life years. Bull World Health Organ.

[5] Murray CJ, Lopez AD, World Health Organization (1996). The global burden of disease: a comprehensive assessment of mortality and disability from diseases, injuries, and risk factors in 1990 and projected to 2020: summary.

[6] Melse JM, Essink-Bot ML, Kramers PG, Hoeymans N (2000). A national burden of disease calculation: Dutch disability-adjusted life-years. Dutch burden of disease group. Am J Public Health.

[7] Budke CM, Jiamin Q, Zinsstag J, Qian W, Torgerson PR (2004). Use of disability adjusted life years in the estimation of the disease burden of echinococcosis for a high endemic region of the Tibetan plateau. Am J Trop Med Hyg.

[8] McKenna MT, Michaud CM, Murray CJ, Marks JS (2005). Assessing the burden of disease in the United States using disability-adjusted life years. Am J Prev Med.

[9] Johnell O, Kanis JA (2006). An estimate of the worldwide prevalence and disability associated with osteoporotic fractures. Osteoporos Int.

[10] Naghavi M, Abolhassani F, Pourmalek F, Lakeh MM, Jafari N, Vaseghi S (2009). The burden of disease and injury in Iran 2003. Popul Health Metr.

[11] Aikins ADG, Unwin N, Agyemang C, Allotey P, Campbell C, Arhinful D (2010). Tackling Africa's chronic disease burden: from the local to the global. Glob Health.

[12] Charlson FJ, Stapelberg NJ, Baxter AJ, Whiteford HA (2011). Should global burden of disease estimates include depression as a risk factor for coronary heart disease?. BMC Med.

[13] Kearns K, Dee A, Fitzgerald AP, Doherty E, Perry IJ (2014). Chronic disease burden associated with overweight and obesity in Ireland: the effects of a small BMI reduction at population level. BMC Public Health.

[14] Murray CJ, Vos T, Lozano R, Naghavi M, Flaxman AD, Michaud C (2012). Disability-adjusted life years (DALYs) for 291 diseases and injuries in 21 regions, 1990–2010: a systematic analysis for the global burden of disease study 2010. Lancet.

[15] Soerjomataram I, Lortet-Tieulent J, Parkin DM, Ferlay J, Mathers C, Forman D (2012). Global burden of cancer in 2008: a systematic analysis of disability-adjusted life-years in 12 world regions. Lancet.

[16] Murray CJ, Barber RM, Foreman KJ, Ozgoren AA, Abd-Allah F, Abera SF (2015). Global, regional, and national disability-adjusted life years (DALYs) for 306 diseases and injuries and healthy life expectancy (HALE) for 188 countries, 1990–2013: quantifying the epidemiological transition. Lancet.

[17] Population Pyramid (2019). Population pyramids of the world from 1950 to 2100 retrieved on 10 march 2020.

[18] Ahmad OB, Boschi-Pinto C, Lopez AD, Murray CJ, Lozano R, Inoue M. Age standardization of rates: a new WHO standard. Vol 9, Issue 10. Geneva: World Health Organization; 2001.

[19] Ghosh S, Arokiasamy P. Morbidity in India trends, patterns and differentials. J Health Stud. 2009:129–40.

[20] Anand S, Hanson K (1997). Disability-adjusted life years: a critical review. J Health Econ.

[21] Reinsdorf MB, Diewert WE, Ehemann C (2002). Additive decompositions for fisher, Törnqvist and geometric mean indexes. J Econ Soc Meas.

[22] Dholakia RH (1986). Removing the residual in standardization procedures. Rev Regional Stud.

[23] Brown AJ (1969). Surveys of applied economics: regional economics, with special reference to the United Kingdom. Econ J.

[24] Global tuberculosis report 2017. Geneva: World Health Organization; 2017. License: CC BY-NCSA 3.0 IGO.

[25] AbouZahr C (2003). Global burden of maternal death and disability. Br Med Bull.

[26] Global Burden of Disease Collaborative Network (2018). Global burden of disease study 2017 (GBD 2017) results.

[27] Indian Council of Medical Research, Public Health Foundation of India, and Institute for Health Metrics and Evaluation (2017). GBD India compare data visualization.

